# Self-Care and Chronic Disease

**DOI:** 10.1155/2013/827409

**Published:** 2013-11-18

**Authors:** Victoria Vaughan Dickson, Robyn A. Clark, Eneida Rejane Rabelo-Silva, Harleah G. Buck

**Affiliations:** ^1^College of Nursing, New York University, 726 Broadway, 10th Floor, New York, NY 10003, USA; ^2^Flinders University, Bedford Park, SA 5042, Australia; ^3^School of Nursing at Federal University of Rio Grande do Sul, Porto Alegre, RS, 90620-110, Brazil; ^4^School of Nursing, Pennsylvania State University, University Park, PA 16802, USA

Chronic diseases are the leading cause of death and disability worldwide. They account for almost 60% of all deaths and 43% of the global burden of disease [1]. This is expected to markedly increase with chronic disease contributing 73% of all deaths and 60% of the global burden of disease by 2020 [1]. The most prevalent chronic diseases—cardiovascular diseases, cancer, and type 2 diabetes—are linked by common and preventable biological risk factors (i.e., high blood pressure, dyslipidemia, and obesity) as well as major behavioral risk factors including unhealthy diet, physical inactivity, and tobacco use. Patient centered care, which includes self-care or self-management, is a fundamental concern for nursing and essential to the prevention and management of chronic diseases [2]. In fact, leading agencies across the globe currently emphasize the importance of patients' self-management of chronic illness symptoms and treatment. This is an international problem requiring international collaboration to address the needs of this at-risk group [3].

In this issue, the international community of nurse scientists and clinicians has contributed to an increased understanding of the multidimensional influences on self-care in chronic disease by describing predictors of self-care and related outcomes and effective strategies to help people with chronic illness improve their health and quality of life. In addition the ongoing gaps in the science of self-care are highlighted.

We are pleased to include the findings of studies from nurse scientists in Australia, Brazil, Iran, Republic of Korea, Portugal, and the United States in the three most common chronic disease populations—cardiovascular disease, cancer, and type 2 diabetes. Topics covered in this special issue include predictors of self-care in Brazilian patients with heart failure following six months of home visits; the influence of health literacy on heart failure knowledge and self-care in the USA; a supportive educational program that improved self-care among Iranian adults with chronic heart failure; and the cross-cultural adaptation and psychometric testing of the Brazilian version of the Self-Care of Heart Failure Index version 6.2. As heart failure reaches epidemic proportions in all parts of the world [4], these papers add to our understanding of the influences of heart failure self-care and help to advance the science by addressing the need for culturally appropriate measurement and interventions.

The importance of providing holistic nursing care to Portuguese patients with cancer is highlighted by S. M. O. Pinto et al. In this study, authors described the importance of religious beliefs, optimism, and being well-informed as contributing to quality of life among patients undergoing chemotherapy and the essential role of nurses throughout the process.

Another global chronic disease on the rise is diabetes. The number of adults with diabetes is expected to increase from 366 million in 2011 to 552 million by 2030 [5]. A. Thomas and A. Ashcroft address an important and understudied population, individuals who undergo acculturation from traditional to modern life style with resulting increased risk for type 2 diabetes. As the global population becomes increasingly mobile, understanding increased risk from changes in lifestyle behaviors like physical activity and dietary choices is increasingly important for all nurses.

Finally, despite the advances in nursing research of self-care and chronic disease over the past few decades, the literature reviews in this special edition remind us that there is still a great deal of conceptual and psychometric work to be done. Self-care in chronic disease is complex and there are significant limitations in the science including issues with precise yet pragmatic measurement of multiple chronic conditions, the need for rigorous scientific methodology, and, most importantly, addressing health equity issues by ensuring adequate representation of diverse populations in our research.

This special edition reminds us that the global nursing community shares common concerns and challenges as well as overarching, common goals. As the number of individuals with chronic diseases like cardiovascular disease, cancer, and diabetes continues to increase, it is imperative that we as nurse scientists and nurse clinicians collaborate to improve the health and quality of life of patients with chronic disease worldwide.

*Victoria Vaughan Dickson* *Victoria Vaughan Dickson* *Robyn A. Clark* *Robyn A. Clark* *Eneida Rejane Rabelo-Silva* *Eneida Rejane Rabelo-Silva* *Harleah G. Buck* *Harleah G. Buck*
